# Evaluating How and Why the Environmental Context Shapes the Course of Development Across the Adult Lifespan

**DOI:** 10.1093/geroni/igab046.1513

**Published:** 2021-12-17

**Authors:** Omar Staben, Frank Infurna, Eileen Crimmins

## Abstract

There is a long-standing literature that has documented the importance of both the immediate and distal context in impacting mental and physical health across the adult lifespan. The goal of this symposium is to bring together a collection of papers that target the extent to which the immediate and distal context as measured through objective and subjective indicators relate to pertinent outcomes of mental and physical health. Staben and colleagues use an intensive longitudinal design in middle-aged adults to show that objective and subjective indicators of the neighborhood are associated with higher levels of and are protective against the impact of monthly adversity on mental health and well-being. Munoz and colleagues evaluate associations between objective and subjective early-life neighborhood contexts and whether they play a role in cognitive function at midlife. They find that poorer age-five self-report conditions were associated with lower working memory. Osuna and colleagues examine how both neighborhood and housing conditions play a role on psychological well-being. They find that housing and neighborhood safety conditions are associated with depressive symptoms over time. Piazza and colleagues examine associations between daily financial thoughts, SES, and indices of emotional and physical health. They find that individuals who reported more daily financial thoughts also reported more negative affect and physical symptoms. The discussion by Crimmins will integrate the four papers by highlighting the importance of how different forms of context can impact development in adulthood and old age, particularly in relation to health and well-being and consider future routes of inquiry.

